# A Newly Discovered Humic-Reducing Bacterium, *Pseudomonas geniculata* PQ01, Isolated From Paddy Soil Promotes Paraquat Anaerobic Transformation

**DOI:** 10.3389/fmicb.2020.02003

**Published:** 2020-09-01

**Authors:** Chunyuan Wu, Xiaoyan Wu, Shanshan Chen, Dongming Wu

**Affiliations:** ^1^Environment and Plant Protection Institute, Chinese Academy of Tropical Agricultural Sciences, Haikou, China; ^2^Hainan Engineering Research Center for Non-point Source and Heavy Metal Pollution Control, Haikou, China; ^3^Danzhou Scientific Observing and Experimental Station of Agro-Environment, Ministry of Agriculture, Danzhou, China

**Keywords:** paraquat, humic-reducing microorganism, *Pseudomonas geniculata*, anthraquinone-2, 6-disulfonate, anaerobic transformation

## Abstract

Due to toxicity and persistence of paraquat (a widely used herbicide), eco-friendly remediation approaches to its contamination and effective antidotes to its poisoning have been highly desired and raised increasing concerns. Paraquat degradation was lesser in aerobic soil in comparison with anaerobic soil, and humic-reducing microorganisms (HRMs) play a key role in paraquat anaerobic transformation process. However, the degradation pathways and related mechanisms remain poorly understood. In this study, we investigated the specific interaction mechanisms of the paraquat transformation processes mediated by a humic-reducing strain under anaerobic conditions. A strain of pure culture, designated as PQ01, was successfully isolated from paddy soil using anaerobic enrichment procedure, and identified as *Pseudomonas geniculata* using phenotypic and phylogenetic analysis. Sucrose, glucose, pyruvate, formic acid, and acetic acid were shown to be favorable electron donors for the reduction of anthrahydroquinone-2,6-disulfonate (AQDS) reduction by PQ01. The strain also had the ability of reducing Fe(III) (hydr)oxides in the presence of sucrose with efficiencies in the order of ferrihydrite > α-FeOOH/γ-FeOOH > γ-Fe_2_O_3_ > α-Fe_2_O_3_. In the “PQ01 + paraquat + AQDS + sucrose” system, AQDS reduction and paraquat biotransformation by strain PQ01 occurred simultaneously, and the presence of sucrose significantly enhanced the biotransformation. Specific mechanisms of the electron transfer processes are promoted by both PQ01 and AQDS, and proceed in two aspects: (1) paraquat served as electron donor in the anaerobic reduction of AQDS by strain PQ01; (2) AQDS was reduced by PQ01 anaerobic metabolism to produce AH_2_QDS, which can directly react with paraquat under anaerobic conditions to generate a single crystal compound (molecular formula of the unit structure is C_2__6_H_2__0_N_2_O_8_S_2_), causing the paraquat to decline dramatically. In conclusion, this main mechanism included the microbial reduction of AQDS to AH_2_QDS, followed by the abiotic reaction between AH_2_QDS and paraquat. This study reported the new characteristics of *P. geniculata* capable of reducing humics analogs, Fe(III) (hydr)oxides, and paraquat, and proposed a novel electron transformation mechanism of the HRMs’ mediated degradation of organic contaminants.

## Introduction

Humic-reducing microorganisms (HRMs) are able to use humic substances as an electron acceptor for the anaerobic oxidation of organic compounds and hydrogen, and ubiquitous in anaerobic soil and sediments (Lovley et al., 1996; Van Trump et al., 2006). For the past 30 years, the interactions between HRM and organic or metal contaminants have been extensively studied (Lovley et al., 1996; Zhao et al., 2019). Besides the important role in humic substance (humics) reduction to generate reducing active substance, HRM can also dynamically involve in the transformation of organic contaminants and metals in anaerobic environments (Field and Cervantes, 2005; Bhushan et al., 2006; Belchik et al., 2011; Wu et al., 2018). Depending on the microbial metabolism of HRM, the organic contaminants are biodegraded either as an electron donor (vinyl chloride, dichloroethene, phenanthrene, etc.) (Bradley et al., 1998; Ma et al., 2011) or as an electron acceptor [carbon tetrachloride, azo dyes, 1,1,1-trichloro-2,2-bis(4-chlorophenyl) ethane, etc.] (Cervantes et al., 2004; Hong et al., 2007; Wu et al., 2014). In addition, reduced humics, mainly generated from humics microbial reduction, can transfer electrons to reducible pollutants (pentachlorophenol, 2,4-dichlorophenoxyacetic acid, arsenic, uranium) to enhance their degrading rate in anaerobic environments (Lovley et al., 1998; Wu et al., 2013b; Qiao et al., 2019; Zhao et al., 2019). Quinone moieties (anthraquinone-2,6-disulfonate, AQDS) have been comprehensively examined as humics-like substances and implicated as the redox-active main groups accepting the electrons. In recent years, HRM and humics-mediated reduction of extracellular substrates have garnered great attention, and more than a hundred HRMs have been isolated from a broad diversity of environments (Gralnick and Newman, 2007; Wang et al., 2009; Zhao et al., 2019). Due to the importance of HRM in the transformation of pollutants and less than 1% of the microorganisms in the environment can be cultured, the discovery of HRM resources is still a research focus in the field of environmental microbiology.

Paraquat (1,1′-dimethyl-4,4′-bipyridylium dichloride) is a cationic non-systemic contact herbicide that has been widely applied for more than 50 years in pre-plowing spraying and zero-tillage paddy fields. Despite its agricultural benefits, the herbicide has high toxicity to humans (Ko et al., 2017) and can result in serious ecological issues after using in agriculture such as adverse effect on blood parameters of fish (Ogamba et al., 2011; Hashemi et al., 2017), teratogenic effects on developing frog embryos (Osano et al., 2002), and inhibition on soil microbial diversity and green algae (Othman et al., 2012; Zhang et al., 2013). In addition to its hazards, paraquat does not readily degrade in natural environments and its half-life in soils varies between 1.3 and 13 years (Rashidipour et al., 2019). Owing to its persistence and toxicity, paraquat has been banned in some countries, including Austria, South Korea, and the European Union; however, it is still being used in more than 90 countries, including China, Tailand, Brazil, Australia, and the United States (Cha et al., 2016; Ko et al., 2017; Huang et al., 2019; Zhang et al., 2019). Considering its extensive applications and hazards on environments and humans, eco-friendly means of remediating paraquat contamination and effective antidotes to paraquat poisoning are highly desired and raised increasing concerns (Yu et al., 2012; Gao et al., 2018; Huang et al., 2019; Zhang et al., 2019).

Microbial degradation is a significant pathway for paraquat detoxification and is eco-friendly. It requires isolating the high-efficiency paraquat-degrading microorganisms. Hitherto, a few paraquat-degrading microorganisms have been isolated from soil and characterized (Huang et al., 2019), and most of these species have been identified as aerobic microorganisms that promote the aerobic degradation of paraquat (Huang et al., 2019). However, according to Bandara and Kearney (1991), paraquat degradation was faster in anaerobic soil in comparison to aerobic soil, and organic matter enhanced and facilitated microbial degradation of paraquat under anaerobic conditions. Subsequently, Wu et al. (2013a) reported the successful and almost complete anaerobic degradation of paraquat (50 mg/L) by three facultative anaerobic HRMs isolated from vegetable soil. In the presence of the quinone AQDS and sucrose, the herbicide was degraded within 5 days, resulting in a significantly higher degradation rate than those determined under aerobic conditions. Overall, aforementioned studies suggest that HRM may be used to effectively eliminate paraquat from soil environments; however, the mechanism implicated in the degradation process remains unclear.

Based on the significant research value on both humic-reducing bacteria and remediation approaches or antidote to paraquat contamination, we conducted a study to reveal the specific interaction mechanisms of the paraquat degradation processes mediated by a humic-reducing strain under anaerobic conditions. The specific objectives were to (1) isolate, characterize, and identify an effective humic-reducing strain PQ01; (2) determine its optimum cultivated conditions and electron donors; (3) explore its potential capacity of reducing humics and Fe(III) (hydr)oxides; and (4) investigate the anaerobic transformation pathway of paraquat by PQ01. This study may be informative to understand the environmental functions of HRM involving in organic pollutants and provide new insight into paraquat anaerobic microbial transformation processes.

## Materials and Methods

### Chemicals

All reagents used in this study, including sodium bicarbonate, ammonium chloride, sodium dihydrogen phosphate, and potassium chloride, were of analytical reagent grade and used without further purification. Biotin, folic acid, pyridoxine hydrochloride, and other vitamins were purchased from Beijing Braun Granville Technology Co. (China). Beef extract, peptone, agar powder, and yeast extract powder were purchased from Guangzhou Chemical Reagent Co. (China). Ferrihydrite was prepared using a standard method adapted from Roden and Urrutia (2002). Goethite (α-FeOOH) was synthesized according to the procedures of Li et al. (2008). Preparation of lepidocrocite (γ-FeOOH), hematite (α-Fe_2_O_3_), and magnetite (γ-Fe_2_O_3_) followed the method described by Li et al. (2007). Humics analogs [anthraquinone-2,6-disulfonate (AQDS), anthraquinone-2-disulfonate (AQS), and anthraquinone-2-carboxylic acid (AQC)] of chemical grade were purchased from Sigma-Aldrich (Tokyo, Japan). Paraquat and sodium 1-heptanesulfonate of analytical grade were purchased from Accu Standard (New Haven, United States). All stock solutions were prepared using deionized water.

### Enrichment and Isolation

According to previous studies, AQDS has been used extensively as a humics analog in studies on humics for microbial respiration (Lovley et al., 1996; Lipczynska-Kochany, 2018). The inoculum source for enrichment was the paddy field in Hainan province, China. Five grams of soil samples was added to 100 ml of anaerobic liquid medium containing mineral salts medium (MSM), 5 mmol/L electron donor, 0.5 mmol/L AQDS (electron acceptor), and 10 mmol/L carbonate buffer. The preparation of MSM was performed according to Ma et al. (2011). Primary enrichment was initiated in anaerobic culture serum bottles (purged with O_2_-free N_2_ for 15 min, sealed with a butyl-rubber stopper and aluminum cap). Upon enrichment, the color of the bottle contents changed to orange, and the enriched population was serially diluted and plated onto Luria–Bertani (LB) medium (10 g/L peptone, 10 g/L NaCl, 5 g/L yeast extract, pH 6.5) at 30°C for 48 h. Afterward, distinct colonies were picked and streaked three times on agar plates, and each colony was tested in sterilized anaerobic medium. All bottles were incubated in the dark at 30°C, the optimal temperature for the growth of the PQ01 microbial strain.

The effects of culture medium [beef extract peptone medium (NA), raw medium (SB), and yeast peptone glucose medium (YPG)], temperature (4, 25, 30, 37, 45, and 60°C), pH (4, 5, 6, 7, 8, 9, and 10), and NaCl concentration (0.5, 1, 2, 3, 5, 7, 10, 12, 15, or 20%) on PQ01 growth were analyzed by varying these parameters.

### Phenotypic and 16S rRNA Analysis

The physiological and biochemical characteristics of the isolated microorganism were determined via the standard methods (Buchanan and Bergey, 1984). The 16S rRNA gene of the isolate was amplified by PCR as described previously (Ma et al., 2011). The obtained sequences were then aligned with related 16S rRNA sequences from GenBank data libraries^1^ and the Ribosomal Database Project^2^. Corresponding nucleotide sequences of representatives of the genus *Pseudomonas* were aligned using the program CLUSTAL X (Thompson et al., 1997). The phylogenetic tree was constructed with the software package MEGA version 6.02 using the maximum-parsimony and neighbor-joining method according to Kimura two-parameter model and bootstrap analyses based on 1000 replicates.

### Batch Anaerobic Incubation Experiments

All reduction experiments were conducted in 25-ml serum bottles under anaerobic and sterile conditions, using the resting cell suspension of PQ01. The suspension was aerobically prepared in LB medium at 30°C, and then harvested in the late log phase by centrifugation (8000 × *g* at 4°C for 10 min). The pellets were washed twice before being re-suspended in fresh sterile MSM. The optical density of the suspensions was adjusted to about 1.5 (λ = 600 nm) (Wu et al., 2014).

To investigate the effect of alternative electron donors on AQDS (0.5 mmol/L) reduction, 12 types of organic substrates (5 mmol/L) were tested, including cellulose, acetic acid, formic acid, propionic acid, toluene, ethanol, glycerol, glucose, sucrose, lactic acid, formic acid, and pyruvate. The medium without cells or organic substrates served as the control set.

To test the alternative electron acceptors for PQ01 anaerobic metabolisms, each bottle contained 1 ml cells, 20 ml MSM (pH 6.5) with 5 mmol/L sucrose (electron donor), and one of the following substrates as electron acceptor: 0.5 mmol/L of AQDS, AQS, and AQC; 25 mmol/L of ferrihydrite, α-FeOOH, γ-FeOOH, α-Fe_2_O_3_, and γ-Fe_2_O_3_. Two control assays were performed under the same conditions: an abiotic set without bacterial cells and a biotic set without the addition of sucrose. All experiments were conducted in triplicate.

### Paraquat Anaerobic Transformation Experiments

All paraquat transformation experiments were conducted in culture medium (described previously) containing 50 mg/L paraquat, in combination with 0.5 mmol/L AQDS, 1 × 10^7^ cells/ml (PQ01), and 5 mmol/L sucrose (initial pH of 6.5). The cultures were carried out in 25-ml serum bottles, and the medium was bubbled with N_2_/CO_2_ (80:20) gaseous mixture and filter-sterilized (0.22 μm filters) before inoculation. The vials were sealed with butyl-rubber plugs that were held in place using aluminum caps and incubated at 30°C. All experiments were conducted in triplicate. Three control assays were performed under the same conditions: one abiotic control set with no cells, and two biotic control sets with no electron donor (sucrose) or acceptor (AQDS).

### Analysis Methods

Triplicate bottles were used for chemical analysis at every interval time. The concentrations of AH_2_QDS, AH_2_QS, and AH_2_QC were quantified using a UV–vis spectrophotometer (UV-3600, Tokyo, Japan); the specific wavelengths of AH_2_QDS, AH_2_QS, and AH_2_QC were 408, 397, and 380 nm, respectively (Wu et al., 2010). The total content of Fe(II), including dissolved and sorbed Fe(II), was quantified photometrically at 510 nm after extraction with 0.5 mol/L HCl for 1.5 h and reaction with 1,10-phenanthroline.

The concentration of paraquat was quantitatively determined by high-performance liquid chromatography (Dionex Ultimate-3000, Sunnyvale, United States). Before analysis, the samples were centrifuged at 4000 × *g* at 4°C for 10 min and filtered with 0.22 μm polyvinylidene fluoride micropore membranes. A reverse-phase C_18_ column packed with 5 μm particles (4.6 × 250 mm) was used. The injection volume was 10 μl. The mobile phase was an aqueous solution of 15 mmol/L phosphate buffer solution (pH 2.5, adjusted by triethylamine), mixed with acetonitrile at a ratio rate of 90:10 (*v*/*v*). The flow rate of the mobile phase was 1 ml/min. The wavelength of maximum adsorption of paraquat was 257 nm.

The crystalline structure of the insoluble product was analyzed by X-ray powder diffraction (Ultima IV; Rigaku, Japan) in the 2θ angle range of 5°–90°. The sampling distance was kept at 0.2°/min.

## Results

### Identification of the Strain PQ01

The vegetative cells of the isolated strain PQ01 were rod-shaped, 0.4–0.8 μm in diameter. The cells occurred singly or in pairs, motile at 25°C but not at 30 and 37°C, stained Gram-negative in both the exponential and stationary growth phases. After 48-h incubation on the LB agar plate, the colonies were wet state, neat edge, and with a distinct smooth, transparent morphology.

**FIGURE 1:**
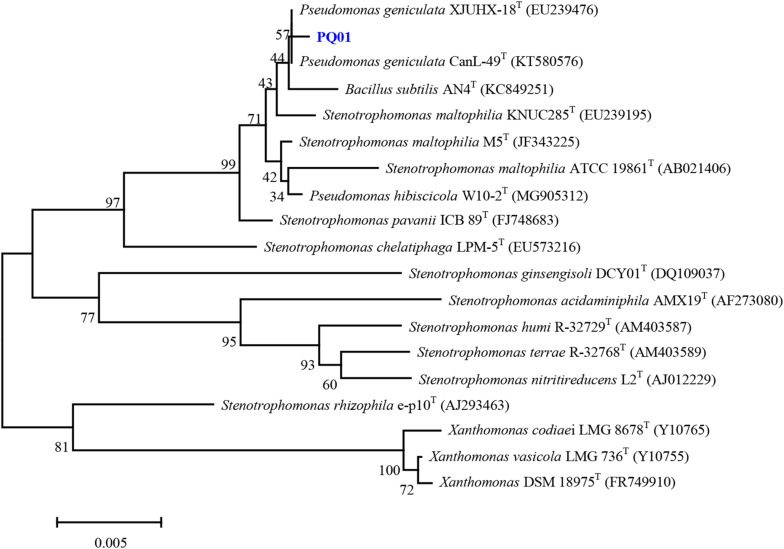
Phylogenetic tree obtained by neighbor-joining analysis based on 16S rRNA gene sequences, showing the position of strain PQ01 among its phylogenetic neighbor. Bar = 0.0005 substitutions per nucleotide position.

The optimum growth temperature of the strain ranged from between 25 to 37°C, and no growth was observed at 45°C and below 5°C. At pH 4.0, the strain did not grow; however, it grew at all other investigated pH conditions (5.0–10.0). The optimum pH for growth was 6.0–9.0. The strain survived at NaCl concentrations of 0–5%, but not at 10% NaCl. Compared with other closely related species, PQ01 is more similar to the N1 strain of *P. geniculata*, as both strains share the positive characteristics of catalase, nitrate reduction, gelatin hydrolysis, and citrate and glucose utility. In addition, the following characteristics were negative for both PQ01 and N1: ornithine decarboxylase, hydrogen sulfide, acid production from glycerol, mannitol, lactose, and rhamnose (Table 1). In terms of overall phenotype characteristics, this isolate should resemble *Pseudomonas*. Furthermore, chemotaxonomic characteristics of the strain and the topology of the 16S-rRNA-based phylogenetic tree (Figure 1) clearly indicated that the nearest phylogenetic neighbors (sequence similarity values ranged from 96.3 to 99.3%) of the strain PQ01 were members of the genus *Pseudomonas*, and the strain had the highest similarity of 99.3% with *P. geniculata* CanL-49^T^ (KT580576). Consequently, the strain was identified as *P. geniculata* strain PQ01, which was deposited in China Center for Type Culture Collection (CCTCC No. M2014107). The nucleotide sequence data for the isolate PQ01 have been deposited in the GenBank nucleotide sequence database under the accession number MT555322.

**TABLE 1:** Characteristics that can be used to differentiate strain PQ01 from the closely related species.

**Characteristic**	**1**	**2**	**3**	**4**	**5**
Catalase activity	+	+	+	+	+
Oxidase activity	–	+	–	–	–
Nitrate reduction	+	+	+	–	–
Hydrolysis of gelatin	+	+	–	+	+
Lysine decarboxylase	–	+	ND	ND	–
Ornithine decarboxylase	–	–	ND	ND	–
Hydrogen sulfide	–	–	–	–	–
Starch red	–	ND	+	+	ND
**Carbon source utilization**					
Citrate utility	+	+	+	+	+
Glucose	+	+	+	+	–
Sucrose	+	–	ND	ND	–
Sorbitol	+	–	–	–	–
Glycerol	–	–	ND	ND	+
Mannitol	–	–	+	–	+
Lactose	–	–	+	–	–
Rhamnose	–	–	–	+	–

**1. P. geniculata PQ01; 2. P. geniculata N1 (data from Liu et al., 2019); 3. P. nitroreducens H45 (Huang et al., 2012); 4. P. hibiscicola JQ291604 (Huang et al., 2012); 5. P. graminis DSM 11363^T^ (Behrendt et al., 1999); +, positive; –, negative; ND, not determined.**

### Electron Donors for AQDS Reduction

We investigated the optimal cultivating conditions for AQDS reduction by the isolate PQ01 and if it can grow during anaerobic metabolism. Twelve types of organic substrates were tested as the alternative electron donors for AQDS reduction by PQ01.

**FIGURE 2:**
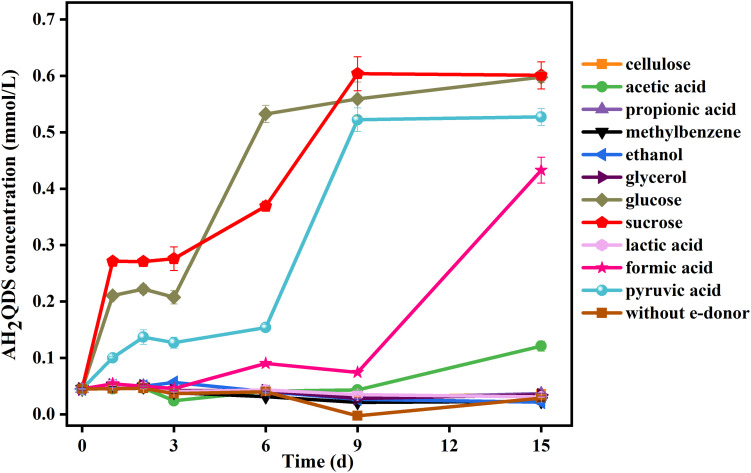
Effect of electron donors (5 mmol/L) on AQDS (0.5 mmol/L) reduction by strain *P. geniculata* PQ01. The experiments were conducted in the medium at pH 6.5 and were performed under anaerobic conditions at 30°C in the dark. Error bars represent the SDs of the means (*n* = 3).

The results (Figure 2) showed that the AQDS was not significantly reduced in the incubations with cellulose, propionic acid, toluene, ethanol, lactic acid, and glycerol. After 15 days, the production of AH_2_QDS increased with time in the incubations with glucose, sucrose, acetic acid, formic acid, and pyruvic acid. Among these species, glucose, pyruvate, and sucrose presented the highest capacity to donate electrons because they could induce the generation of AH_2_QDS after only 7 days of incubation. Formic acid and acetic acid required a longer time (approximately 9 days) to trigger AQDS reduction. The control sets having no PQ01 or no organic substrates did not show any AH_2_QDS (data not shown here).

These results indicate that glucose, sucrose, acetic acid, formic acid, and pyruvic acid could serve as favorable electron donors for AQDS reduction by strain PQ01. The electron-donating efficiency of these compounds decreases in the order of sucrose > glucose > pyruvate > formic acid > acetic acid. Other organic substrates such as cellulose, lactic acid, propionic acid, toluene, ethanol, and glycerol cannot be used as electron donors for the reduction of AQDS.

### Alternative Electron Acceptors

**FIGURE 3:**
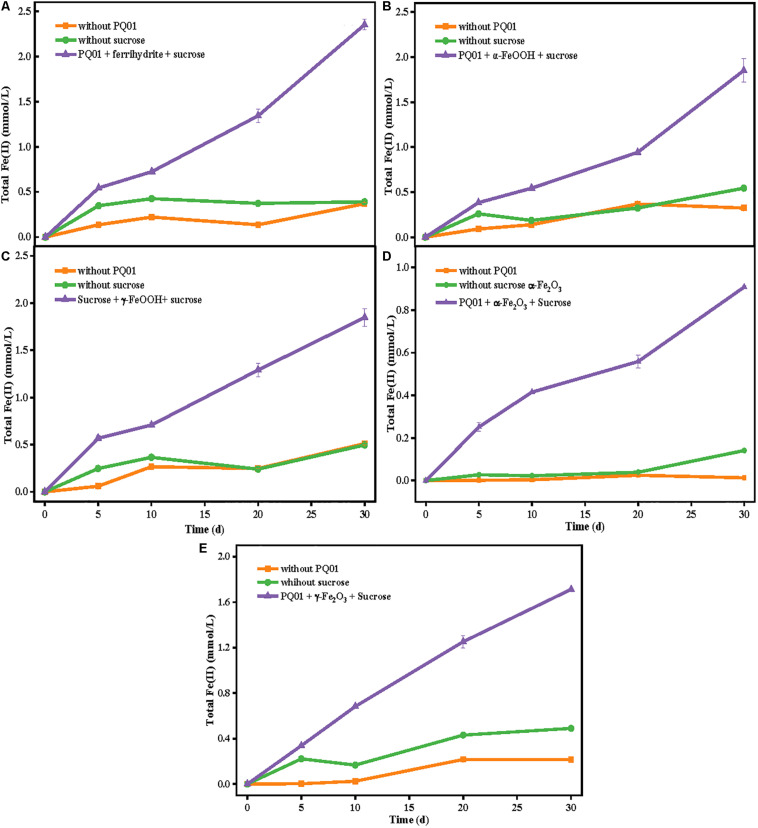
The production of total Fe(II) from the reduction of 25 mmol/L ferrihydrite **(A)**, α-FeOOH **(B)**, γ-FeOOH **(C)**, α-Fe_2_O_3_ **(D)**, or γ-Fe_2_O_3_ **(E)** by the strain PQ01 using 5 mmol/L sucrose as the electron donor. The experiments were performed under anaerobic conditions at 30°C for 30 days. Error bars represent SDs of the means (*n* = 3).

Using a similar experimental procedure, microbial reduction of Fe(III) by strain PQ01 was explored with five types of Fe(III) (hydr)oxides: ferrihydrite, α-FeOOH, γ-FeOOH, α-Fe_2_O_3_, and γ-Fe_2_O_3_. As shown in Figure 3, after 30 days, less Fe(II) was formed in the biotic control without sucrose or active cells. In each reacting system, total Fe(II) concentration increased with the incubation time, varied in the order of ferrihydrite (2.3 mmol/L) > γ-FeOOH (1.8 mmol/L) = α-FeOOH (1.8 mmol/L) > γ-Fe_2_O_3_ (1.7 mmol/L) > α-Fe_2_O_3_ (0.9 mmol/L). These results suggest that Fe(III) reduction by PQ01 is a biological process as it requires both active cells and sucrose, and all the five types of Fe(III) oxides can be reduced by the strain with sucrose as the electron donor, and the reduction rate of ferrihydrite is the highest, followed by γ-FeOOH,α-FeOOH, γ-Fe_2_O_3_, and α-Fe_2_O_3_.

**FIGURE 4:**
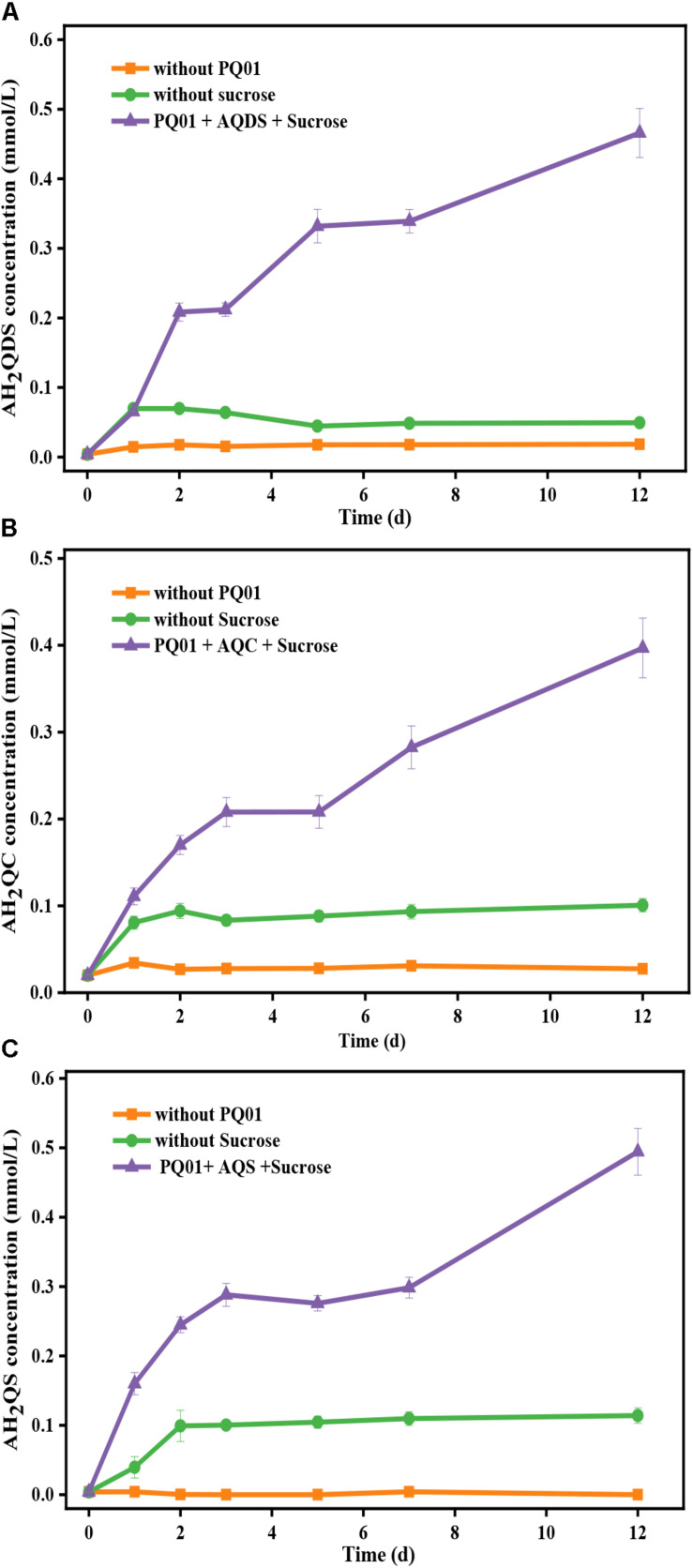
The reduction of 0.5 mmol/L humics analogs by *P. geniculata* PQ01 with 5 mmol/L sucrose as the electron donor: **(A)** AQDS, **(B)** AQC, and **(C)** AQS, respectively. The experiments were performed under anaerobic conditions at 30°C for 12 days. Error bars represent the SDs of the means (*n* = 3).

The reduction of the three humics analogs AQDS, AQC, and AQS serving as the electron acceptor by strains PQ01 using sucrose as the electron donor is shown in Figure 4. After a 12-day incubation, the concentration of quinones in the controls without sucrose (biotic control) or active cells (abiotic control) remained almost unchanged, demonstrating that quinones were persistent in the absence of microbial activity of PQ01 and the chemical reduction of quinones by sucrose was negligible. In contrast, quinones were significantly reduced in the active treatments (sucrose + quinones + PQ01), and the concentration of AH_2_QDS, AH_2_QC, and AH_2_QS reached 0.46, 0.40, and 0.49 mmol/L, respectively. These results suggest that AQDS, AQS, and AQC are able to serve as favorable electron acceptors in the anaerobic metabolism of PQ01, and the reducing capability of strain PQ01 for quinones ranks as AQS > AQDS > AQC.

### Paraquat Anaerobic Transformation by Strain PQ01

**FIGURE 5:**
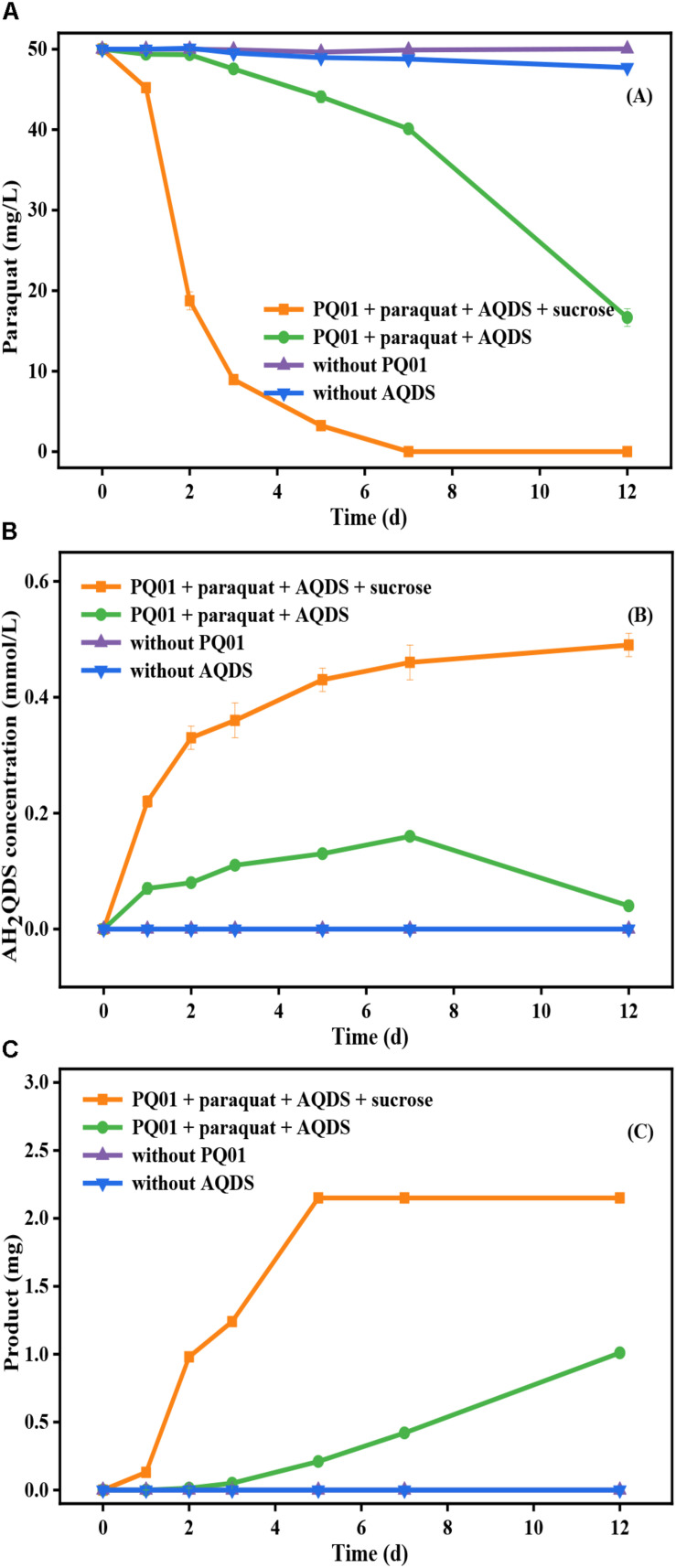
Anaerobic transformation of paraquat by *P. geniculata* PQ01 during 12-day incubation: **(A)** paraquat; **(B)** AH_2_QDS; **(C)** insoluble product. The experiments were performed at 30°C using 50 mg/L paraquat, 0.5 mmol/L AQDS, 5 mmol/L sucrose, and 1.00 ml cell suspension (1.00 × 10^7^). Error bars represent the SDs of the means (*n* = 3).

Further, the anaerobic transformation of paraquat by strain PQ01 was studied. As shown in Figure 5, the concentration of paraquat in the treatment set of “PQ01 + paraquat + AQDS” decreased from 50 to 16.7 mg/L within 12 days. At the end of the incubation period, 1 mg of irregularly shaped, dark green, long crystals was collected (Figure 5C). When sucrose (electron donor for AQDS reduction) was added in the system, paraquat transformation was significantly enhanced; almost no paraquat was detected after 7 days of incubation (Figure 5A). Meanwhile, insoluble crystals of 2.15 mg with dark green color were generated from the system (Figure 5C). In the control sets without PQ01 or AQDS, the concentration of paraquat remained unchanged and no crystals were observed, suggesting that paraquat reduction depended on both PQ01 and AQDS. Further, after a 12-day incubation, the reduction of AQDS was observed in the treatments of “PQ01 + paraquat + AQDS” and “PQ01 + paraquat + AQDS + sucrose,” and the concentration of the reduction product AH_2_QDS was increasing during the first 7-day incubation, whereas the concentration of AQDS in the treatment without active cells (paraquat + AQDS + sucrose) remained almost unchanged.

All the aforementioned results demonstrated that the AQDS microbial reduction and the paraquat microbial anaerobic transformation took place simultaneously in the treatments of “PQ01 + paraquat + AQDS” and paraquat was able to serve as electron donor in the anaerobic reduction of AQDS by strain PQ01. Interestingly, in this treatment, paraquat concentration declined dramatically from 40.11 to 16.7 mg/L after 7 days of incubation, when the AH_2_QDS concentration began to decrease from 0.16 to 0.04 mmol/L (Figures 5A,B). From these results, it can be deduced that the main decrease of paraquat was closely related to the AH_2_QDS decrease. This hypothesis also explains why there is more paraquat decrease in “PQ01 + paraquat + AQDS + sucrose” treatment, owing to the fact that more AH_2_QDS was produced by PQ01 when sucrose served as the electron donor.

**FIGURE 6:**
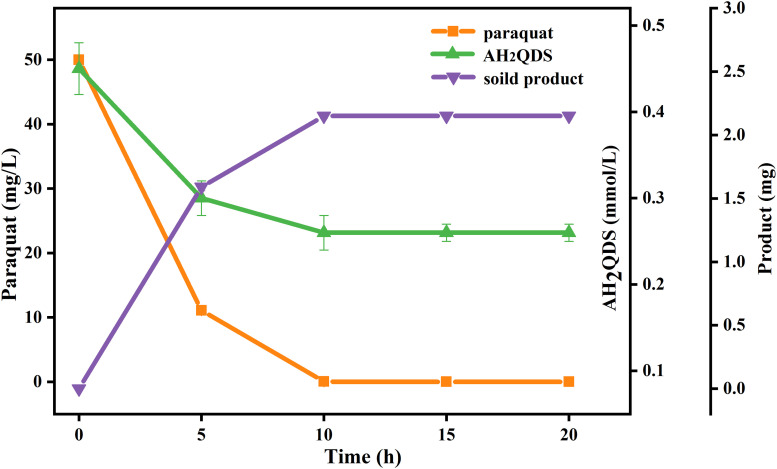
Effect of biogenic AH_2_QDS on anaerobic transformation of paraquat. Paraquat was added to a sterilized and filtered 12-day incubation mixture of *P. geniculata* PQ01, 0.5 mmol/L AQDS, and 5 mmol/L sucrose, and incubation was followed for 20 h to record the decrease in AH_2_QDS and paraquat, and the formation of the crystalline product. Error bars represent the SDs of the means (*n* = 3).

To clarify the probable abiotic reaction between paraquat and AH_2_QDS produced by PQ01, a supplementary experiment was conducted, in which the system of sucrose/cells/AQDS was firstly operated under the same conditions as previous tests and autoclaved after 12-day incubation. Then paraquat was introduced into the system that was incubated in the anaerobic station for another 24 h. As seen in Figure 6, the concentration of paraquat was decreased by nearly 100% after 24 h of incubation, and 2.15 mg of insoluble crystals was formed. Moreover, the amount of AH_2_QDS produced during the first incubation period decreased from 0.45 to 0.26 mmol/L after the second incubation. Considering that the heat-killed cells of PQ01 (killed by autoclaving) and the medium itself cannot react with paraquat, the paraquat transformation in the latter incubation seems to be directly related to the chemical reductive activity of biogenic AH_2_QDS.

**FIGURE 7:**
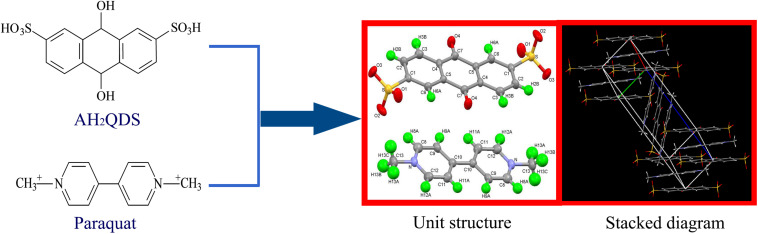
Single-crystal XRD structures of the insoluble product formed via the chemical reaction between paraquat and biogenetic AH_2_QDS.

According to single crystal diffraction analysis (Figure 7), the insoluble product has a monoclinic crystal structure. The unit structure of the crystal is C_2__6_H_2__0_N_2_O_8_S_2_, and it is composed of two basic units, 1-1-dimethyl-4-4-bipyridine and 2,6-disulfonic acid-anthraquinone. These two units have a parallel structure, and they are linked by O–H and C–O bonds.

**FIGURE 8:**
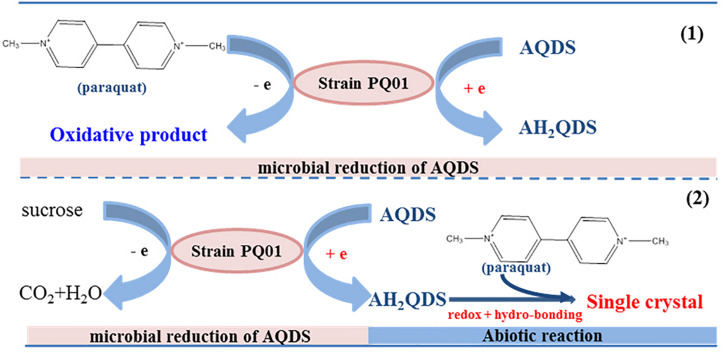
The proposed mechanism of paraquat anaerobic transformation in presence of *P. geniculata* PQ01, AQDS, and sucrose.

Therefore, a mechanism of paraquat anaerobic transformation in the system of humic microbial reducing by PQ01 was proposed, as shown in Figure 8. The humic-reducing strain PQ01 and AQDS were involved in paraquat anaerobic transformation, and proceed in two mechanisms: (1) paraquat serves as an electron donor for PQ01 anaerobic metabolism coupling with extracellular AQDS reduction; (2) biogenetic AH_2_QDS served as the electron donor and reacted with the pyridinium ions of paraquat to form a planar structure that aggregates into insoluble single crystals. The two nitrogen-containing heterocycles of paraquat and the two sulfonic acid groups of AH_2_QDS are evident in the planar structures. The low steric hindrance between these structures facilitates their aggregation, leading to single crystals with a chain confirmation.

## Discussion

The interactions between humics and microorganisms have been researched for the past 30 years. The available studies have suggested that humics may be dynamically involved in the flow of carbon and electron in anaerobic environments (Lovley et al., 1996; Gralnick and Newman, 2007; Zhao et al., 2020). Some microorganisms found in soils are able to use humics as an electron acceptor for the microbial oxidation of organic substrates and hydrogen (Van Trump et al., 2006; Gralnick and Newman, 2007; Wu et al., 2009; Zhao et al., 2020). Subsequently, the quinone moieties in humics (e.g., AQDS) have been implicated as the redox active groups accepting the electrons, and most known HRMs are capable of reducing AQDS to AH_2_QDS. Therefore, quinone compounds are good analogs for the function of humics as a terminal electron acceptor (Field and Cervantes, 2005).

*P. geniculata* has much more potential applications in the field of environment protection. As a strain of environmental origin, *P. geniculata* has been reported capable of degrading phenanthrene, T-2 toxin, nicotine, and industrial dyes (Shi et al., 2013; Yang et al., 2013; Liu et al., 2014), and also active in nitrogen fixation, plant-growth promotion, and seedling growth of aged seeds (Zhang et al., 2010; Gopalakrishnan et al., 2015; Liu et al., 2019). In this study, we identify a new anaerobic metabolism of *P. geniculata* PQ01 that can effectively reduce soluble humics analogs and insoluble Fe(III) (hydr)oxides under anaerobic conditions, using glucose, sucrose, acetic acid, formic acid, or pyruvic acid as the electron donor. The electrons are transferred from the organic substrates to the extracellular substrates [AQDS, AQS, AQC, Fe(III)], which served as the terminal electron acceptors and were reductively transformed into reducible form, coupling with the oxidation of organic substrates, which served as the electron donor. To the best of our knowledge, this is a new finding on describing the capacity of *P. geniculata* to reduce humics and Fe(III) (hydr)oxides.

The finding that microorganisms can donate electrons to humics has important implications for the mechanisms by which microorganisms degrade organic contaminant (Field and Cervantes, 2005; Lipczynska-Kochany, 2018). Anaerobic degradation of organic contaminants by HRM occurs via three main ways: (1) the organic contaminant is directly used as the electron acceptor to be reduced by HRM (Bradley et al., 1998; Cervantes et al., 2004; Belchik et al., 2011; Zhao et al., 2019); (2) the contaminant serves as electron donor to be oxidized through extracellular respiration (Bhushan et al., 2006; Hong et al., 2007; Ma et al., 2011); (3) the electrons are transferred from the electron donor to the humics [or Fe(III)] by microbial respiration, then transferred from the reductant [reduced humics or Fe(II)] to the organic contaminants, leading to the degradation of the contaminant and the regeneration of the reduced humics or Fe(II) (Field and Cervantes, 2005; Wang et al., 2009; Lipczynska-Kochany, 2018; Zhao et al., 2020).

In this study, the humic-reducing strain PQ01 and AQDS were involved in paraquat anaerobic transformation, and the main mechanism included the microbial reduction of AQDS to AH_2_QDS by humic-reducing bacterium PQ01, followed by the abiotic reaction between paraquat and AH_2_QDS, where AH_2_QDS is the reactant and cannot be re-oxidized to AQDS. Obviously, the mechanism of paraquat transformation enhanced by AQDS microbial reduction was different from previous studies that AQDS served as electron shuttles.

Paraquat is a persistent toxic herbicide that does not rapidly degrade in the environment, and the residue levels of paraquat in soil is always high over a period of several months. Therefore, paraquat residue and its toxicity to terrestrial and aquatic environments raise serious concerns (Huang et al., 2019). Degradation of paraquat was reported to be lesser in aerobic soil in comparison with anaerobic soil such as lowland rice field, and organic matter can enhance the degradation rate (Bandara and Kearney, 1991). To understand the mechanism of such enhanced reactivity, we investigated the degradation of paraquat in a humics microbial reducing system. Our results show that paraquat degrade well in humics microbial reducing system with AQDS as the sole electron acceptor, and the addition of organic substrate can enhance the degradation; the degradation rate reached 100% in 5 days of incubation (Wu et al., 2013a). However, it is still unclear why organic substrate can enhance the degradation rate of paraquat and what the degradation pathway is. In this study, we reveal the specific mechanisms of the electron transfer processes promoted by both *P. geniculata* PQ01 and AQDS. The main reaction mechanism is that AQDS is reduced by PQ01 anaerobic metabolism to produce AH_2_QDS, which can directly react with paraquat to form a single crystal compound, causing the paraquat to decline dramatically. In conclusion, this mechanism included the microbial reduction of AQDS to AH_2_QDS, followed by the abiotic reaction between paraquat and AH_2_QDS (Figure 8).

To the best of our knowledge, this study constitutes the first report for the paraquat anaerobic biotransformation pathway and related mechanism linked to humics analog reduction by a pure strain. The ability of strain PQ01 to reduce Fe(III), humics, and facilitate paraquat anaerobic transformation makes it one of the few isolates of genus *Pseudomonas* reported able to do so. Studies should be conducted to further confirm the interactive mechanisms among strain PQ01, humics, and paraquat from a new perspective based on the molecular level and determine whether structurally analogous pesticides such as diquat can undergo similar transformation by the PQ01 strain.

## Data Availability Statement

The datasets generated for this study can be found in the online repositories. The names of the repository/repositories and accession number(s) can be found at: China Center for Type Culture Collection (CCTCC No. M2014107).

## Author Contributions

CW designed the work and wrote the manuscript. XW analyzed samples and prepared the figures. DW and SC conducted the experiment. All authors contributed to the article and approved the submitted version.

## Conflict of Interest

The authors declare that the research was conducted in the absence of any commercial or financial relationships that could be construed as a potential conflict of interest.

## Footnotes

1 http://blast.ncbi.nlm.nih.gov/Blast.cgi

2 http://rdp.cme.msu.edu/seqmatch

## Abbreviations

AH_2_QC: anthrahydroquinone-2-carboxylic acid
AH_2_QDS: anthrahydroquinone-2,6-disulfonate
AH_2_QS: anthrahydroquinone-2-disulfonate
AQC: anthraquinone-2-carboxylic acid
AQDS: anthraquinone-2,6-disulfonate
AQS: anthraquinone-2-disulfonate
HRM: humic-reducing microorganisms
humics: humic substances
*P. geniculata*: *Pseudomonas geniculata.*
